# Evolving stratification and biomarker discovery in cancer research with technological advancement of proteomics: 35 years and counting

**DOI:** 10.1042/BSR20253544

**Published:** 2025-10-09

**Authors:** Divyank Mahajan, Shikha Kaushik, Tapasya Srivastava

**Affiliations:** Department of Genetics, University of Delhi South Campus, New Delhi, 110021, India

**Keywords:** biomarker, cancer, mass spectrometry, metastasis, proteomics

## Abstract

Proteome, the molecular product of regulatory diktat of the cellular machinery, predicts the behaviour and progression of cancers. Designing effective molecular therapies based on proteins with comprehensive patient stratification remains the mainstay of every translational research. Research on the proteome involves a) identification of biomarkers that, with utmost sensitivity and specificity, reveal significant insights into the disease state and b) understanding the mechanistic underpinnings and rewiring of cellular signaling pathways that drive a particular cancerous pathology. In this review, we give a comprehensive description of the evolution of mass spectrometer-based methods, including labeling strategies available to study the proteome and post-translational modifications in response to various perturbations. We summarize their utility in understanding complex processes of cancers, advance research on cancer therapy by decoding novel biomarkers, identify therapy resistance drivers, and enhance spatial attributes of tumor microenvironment by single-cell proteomics. Finally, some of the challenges in the currently used methods have been discussed.

## Introduction

### Early days—the prestable isotopes labeling era

**Figure 1:**
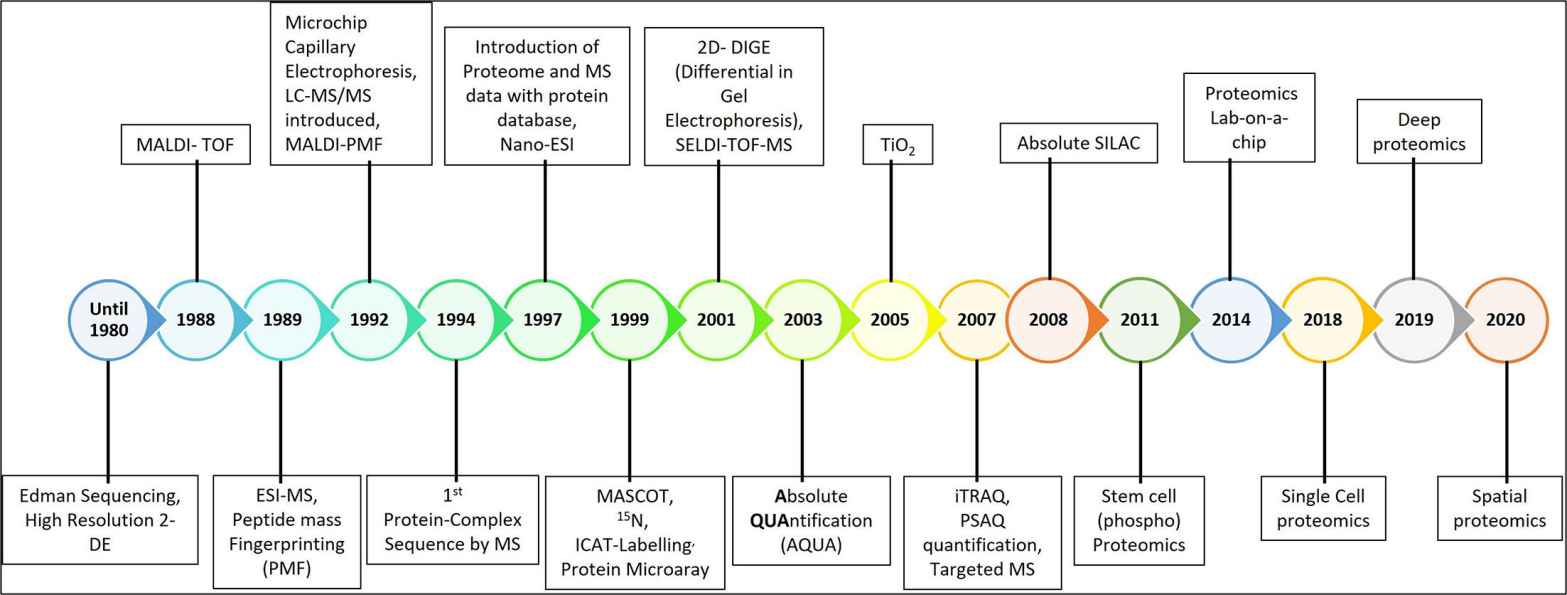
Timeline representing milestones in the evolution of proteomics technology.

The word ‘proteomics’ coined by Mark Wilkins in 1994, referred to the cataloging of all proteins present in a biological system in a given state and time. For this, the complete protein complement of the subject had to be separated using two-dimensional electrophoresis (2DE). Since then, the world of proteomics has evolved significantly as shown in Figure 1, transitioning from basic protein identification to a sophisticated discipline decoding protein interactions, modifications, quantification, and localization. These developments have paved the way for enhanced biomarker discovery and personalized medicine, addressing the complexities of biological samples. In 2D-PAGE, proteins isolated from source samples are separated in the first dimension based on their isoelectric point, known as isoelectric focusing (IEF), and based on their molecular weight in the second dimension using sodium dodecyl sulfate-polyacrylamide gel electrophoresis (SDS-PAGE). Proteins thus resolved are visualized using a protein staining dye such as silver nitrate. Visualized spots of interest are then incised and identified by peptide mass fingerprinting in a mass spectrometer (MS).

Before long, it was realized that a quantitative measure of dynamic changes inside a cell or organism, in response to a genetic, cellular, or environmental perturbation, was critical to understand their biological functioning (e.g. a mutation). Therefore, such measures of change in the relative abundances of proteins have been at the forefront of many proteomics applications, especially in drug discovery. Quantification of proteins initially relied upon 2D-PAGE followed by densitometry analysis to quantify and MS analysis to identify and compare samples, e.g., treated vs. untreated. Incremental changes in the protocols have given many improved versions; however, the method of 2DE-PAGE to characterize changes in the proteome has some critical limitations. For instance, 2DE-PAGE cannot detect various membrane-bound proteins, proteins of low abundance, and proteins with extremely high or very low pI, with high certainty. Therefore, it is practically not possible to be able to observe the full complement of the proteome changes. MS-based proteomics, coupled in line with ultra-thin columns for liquid chromatography (LC), which does not rely on gel-based separation, overcame these caveats, and various new techniques emerged.

### Ionization methods

Ionization is a crucial process in MS, transforming neutral molecules into charged ions for analysis. This process typically involves converting solid or liquid samples into a gaseous state before ionization occurs, often through the loss of an electron, resulting in positively charged cations.

Ionization methods in MS can be broadly categorized into soft and hard ionization techniques, each serving distinct purposes in analytical applications. Soft ionization methods such as electrospray ionization (ESI) [1] and matrix-assisted laser desorption/ionization (MALDI) [2], produce ions with minimal fragmentation that preserve the integrity of the molecular structure, whereas hard ionization methods, including electron impact ionization (EI) and chemical ionization (CI), lead to significant fragmentation, providing detailed structural information [3].

### Soft ionization techniques

#### MALDI (matrix-assisted laser desorption ionization)

MALDI is the first soft ionization technique developed in 1988 [2], which entirely changed the field of analysis of biomolecules such as peptides, proteins, nucleotides, and DNA. Before its inception, established methods of vaporization and ionization proved to be excessively energetic for biopolymers, which are thermo-labile, leading to their fragmentation. This introduced frequent ambiguity in calculating molecular weights and structures of these molecules, an obstacle overcome by mixing the compound to be analyzed in a solvent carrying a special organic compound called matrix. The matrix is typically a small organic molecule having absorption maxima at a laser wavelength, such as a nitrogen laser (337 nm) [4]. After mixing the analyte with the matrix, it is allowed to dry on a probe plate and settle within the crystal lattice of the matrix, thus forming a ‘solid solution’. α-Cyano-4-hydroxycinnamic acid, sinapinic acid (4-hydroxy-3,5-dimethoxycinnamic acid), and 2,5-hydroxybenzoic acid (2,5 DHB) are examples of some frequently used matrix materials. The choice of an appropriate matrix is based on the nature and size of the analyte molecules, and the amount of energy they transfer to the analytes they are co-crystallized with. For example, 2,5 DHB produces minimal interference and turns out to be the best choice for analytes such as high molecular weight proteins and oligosaccharides [5–7], and α-Cyano-4-hydroxycinnamic acid is best suited for tryptic digest produced peptide analytes below 10 kDa [8]. The co-crystallized solution is irradiated with nanosecond pulses of laser, and energy is absorbed by the matrix, which is then transferred to the analyte, causing its protonation. Rapid heating as a result of energy absorption and accumulation by the matrix molecules causes local sublimation of the crystals, eroding a portion of it and forming a plume of gaseous matrix carrying the analyte molecules within it [9]. The formation of singly protonated ions is typical of MALDI; however, the exact mechanism for the formation of the ions is not fully understood. The most widely accepted mechanism suggests that proton transfer from the matrix to the analyte occurs before desorption while in the solid phase, or in the gaseous phase from the photoionized matrix molecules when the matrix-analyte plume is expanding inside the vacuum [10–12].

There are a few particular advantages that MALDI offers over other ionization methods. The foremost includes the option of fully automated data collection and analysis. It is an evolution from spotting of samples over the probe plate (typically a 96-spot plate) done as a separate step prior to MS analysis. This is now performed robotically, thus allowing for the automation of the whole process, including data collection and analysis [13]. Other advantages offered by MALDI for analysis of peptides and proteins are a) using a multi-sample plate offering rapid sample analysis, b) high sensitivity (femto levels), and c) the ability to tolerate heterogeneous samples, such as proteolytic digests d) offering a dynamic analysis range [14]. A drawback of MALDI is the requirement of several hundred pulses of laser shots to achieve an unambiguous signal-to-noise ratio for ion detection. Another major limitation is that it cannot be coupled on-line with LC. It is also limited by low reproducibility between shots. The results also significantly depend on sample preparation methods. Interference of matrix-related ions to mask the signals of interest has been noted when the analyte is below 1000 Da. To overcome various limitations of MALDI, many improved alternatives have been introduced. One set of modifications includes replacing organic matrices with other inorganic materials, such as surface-assisted laser desorption/ionization (SALDI) and desorption/ionization on silicon (DIOS). In SALDI, instead of using the matrix lattice, a surface is applied that absorbs the energy and transfers it to the analyte [15,16].

#### Electrospray ionization

Immediately, a year after MALDI, another ‘soft’ ionization method known as electrospray ionization (ESI) was developed by Fenn et al. [1] to ionize analytes with properties such as those of biomolecules [1]. Similar to MALDI, ESI ionizes labile molecules without any fragmentation using the least residual energy; however, unlike MALDI, it produces ions with multiple charges. This method was rapidly incorporated into the research field and is now the most widely used soft ionization technique for samples in liquid form. In ESI, the ions are formed by releasing samples diluted in a polar volatile solvent via an inlet under an electric field created by applying a potential difference of 3–6kV between the tip of this inlet and the surrounding interface just before entry into the MS. As rapidly as the incoming sample of the capillary encounters the electric field, the linear flow of the stream takes up a conical shape called a Taylor cone, which in itself transforms into a jet spray, causing the dispersion of the sample into highly charged droplets. Once the droplets reach the Rayleigh limit, i.e., the point where repulsion inside the droplet exceeds the surface tension, the ions desorb themselves from the droplet to form bare ions. Because the ions produced are multiply charged, the high molecular weight analytes, such as peptides or even intact proteins, can be observed at far lower mass-to-charge ratios (m/z) on the spectra, and therefore, the molecules with extremely high molecular weights can also be analyzed on MSs with a low mass range.

The biggest advantage that ESI provides over MALDI is the ability to be coupled with liquid chromatographic processes such as HPLC. As a consequence, the sample preparation and fractionation does not need to be done separately before feeding into the ionization and mass analysis. Over time, it has been widely used as a technique to analyze biomolecules of varying polarities whether small or large in complex biological samples such as for proteomics experiments.

### MS-based quantitative proteomics

Proteomics applications in drug discovery mostly require a measure of change in the relative abundances of proteins. Quantification of proteins initially relied upon 2D-PAGE followed by MS analysis to compare two samples, e.g., treated *vs*. untreated. In 2D-PAGE, proteins isolated from source samples are in the first dimension separated based on their isoelectric point, known as isoelectric focusing (IEF), and then based on their molecular weight in the second dimension, using sodium dodecyl sulfate polyacrylamide gel electrophoresis (SDS-PAGE). Separated proteins are then visualized using a protein staining dye such as silver nitrate and quantified using densitometry. Proteins of interest are then identified by cutting out the spot of interest. The method of 2DE-PAGE to characterize proteome changes, however, faces some critical limitations. For instance, 2DE-PAGE cannot detect various membrane-bound proteins, proteins of low abundance, and proteins with extremely high or very low pI are usually missed. Therefore, it is practically not possible to be able to observe the full complement of proteome changes. MS-based proteomics, which does not rely on gel-based separation, overcomes these caveats. In MS-based proteomics, a ‘shotgun’ approach is similar to that in genomics. Protein mixtures to be analyzed are extracted, digested into peptides using a site-specific protease, fractionated, and separated using high-performance LC (HPLC) [17]. In most cases, LC is directly coupled with a mass spectrometer (LC-MS). MS-based proteomics that analyses a peptide mixture is known as ‘bottom-up’ proteomics, contrasting with ‘top-down’ proteomics, which involves analysis of intact proteins. Digested samples fed into the LC are resolved, and based upon the resolving parameter of the LC, such as hydrophobicity, peptides enter the ionization chamber of the MS. Inside the MS, the mass-to-charge ratio (m/z) of ionized peptides is measured and spectra are generated, which are used to identify and quantify proteins. For complex protein mixtures, LC is mostly coupled with tandem MSs, thus making the technology as LC-MS/MS—which helps high-throughput analysis of complex protein mixtures. Further, for MS/MS analysis, mainly two acquisition methods are utilized—data-dependent acquisition (DDA) and data-independent acquisition (DIA). DIA is advantageous over DDA as it provides broader protein coverage, high reproducibility, and accuracy. Using well-standardized pipelines and informatics tools, MS1 and MS2 spectra are analyzed, enabling the identification of thousands of proteins. To identify novel biomarkers or drug targets, quantitative proteomics is concerned with comparing proteome-level changes of two or more conditions. Broadly, there are two approaches to perform quantitative proteomics: *stable-isotope labeling and label-free*. Methods that utilize stable isotopes can further be categorized as metabolic and chemical labeling, discussed below.

### Labeling methods

**Figure 2:**
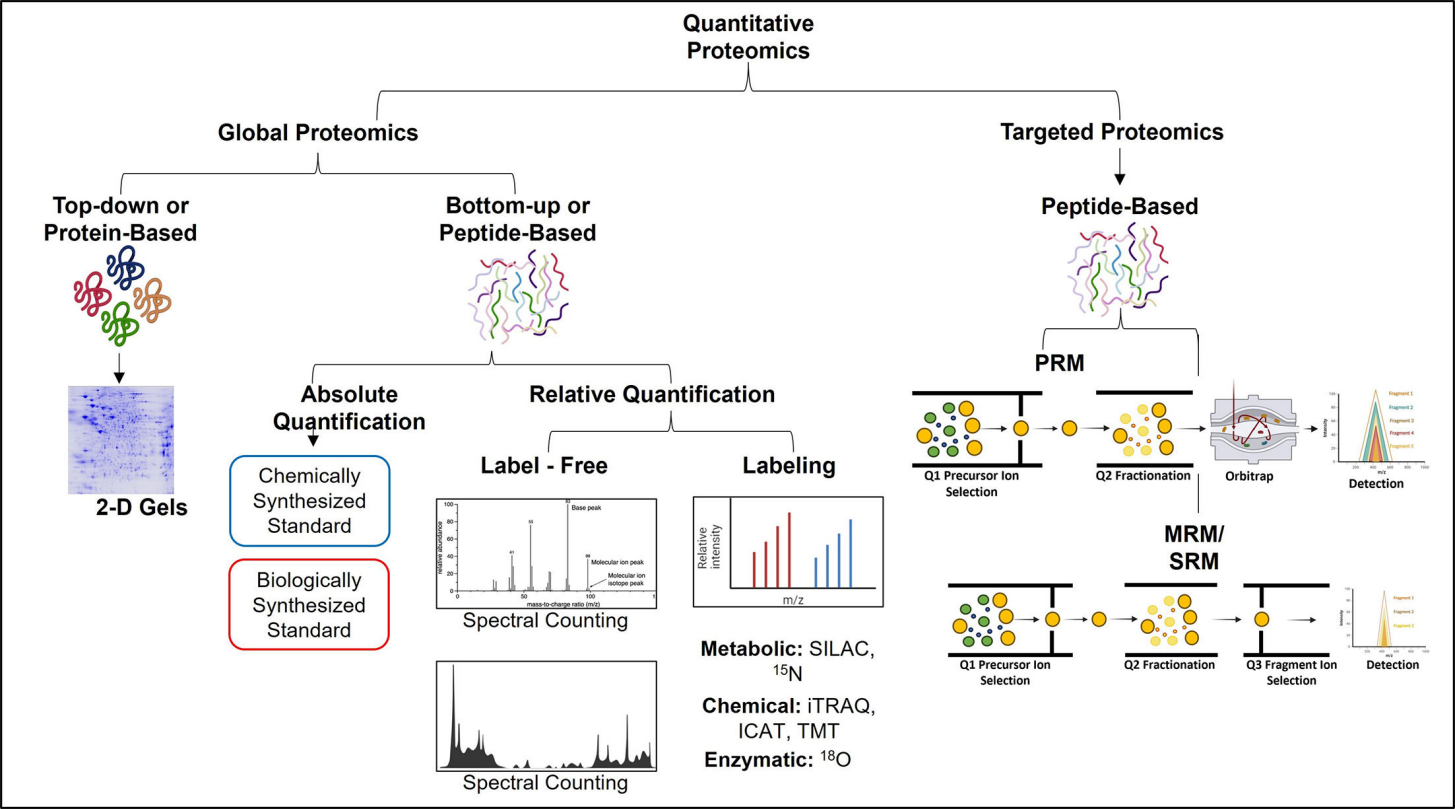
Strategies of quantitative proteomics.

In order to enable improved reproducibility and precise and accurate quantification, labeling reagents can be used to mark the proteins of each sample with different stable isotopes and mixed, thus analyzing the samples to be compared within the same mass spectrum. Labeling with stable isotopes creates a known mass shift between the same peptides from two different samples to be compared within the same MS run and analysis. Labeling of a proteome, usually with isotopes of hydrogen, carbon, and nitrogen, can be performed either metabolically or chemically. One significant advantage that metabolic labeling has over chemical labeling is the incorporation of the labels very early in the timeline of the experiment. This ensures the least variation in the samples to be compared during their preparation. Figure 2 represents the strategies employed for quantitative proteomics to achieve either global or targeted proteomic analyses, each with distinct methodologies and applications. Global proteomics focuses on comprehensive protein profiling, while targeted proteomics hones in on specific proteins of interest, enhancing sensitivity and accuracy.

#### Isotope coded affinity tag—chemical labeling

Aebersold and team [18] developed the first method to chemically label proteins to perform MS-based quantitative proteomics using a novel class of reagents named Isotope Coded Affinity Tags (ICAT) [18]. This was clearly a pioneering method in the field of quantitative proteomics, and in retrospect, proved to be like the industrial revolution of proteins, an impactful change that separates two different times in the history of proteomics. The method involves *in vitro* chemical labeling of proteins with ICAT reagents at the cysteine residues. In order to create a mass difference for differentiating between the same protein peptide but belonging to different samples, a mass difference or shift is added onto the peptides of one of the samples with respect to the other, e.g. normal vs. diseased. ICAT reagents are made up of three functional elements: an affinity tag, a stable isotope-coded linker, and a specific chemical reactor. Of the three, the functional element which provides the requisite mass differential is the middle-isotopic linker chain. In this strategy, after the experimental perturbation, lysate from cellular state-1 is labeled with light ICAT and from cellular state-2 is labeled with heavy ICAT, via the reactive group on the tag and thiol groups on cysteine residues on proteins. The two labeled states are combined and proteolyzed using a site-specific enzyme and, thereafter, using affinity tags available, such as biotin, the tagged proteins are isolated. The isolated peptides are optionally fractionated and analyzed by LC-MS/MS. Every peptide (representative of a protein) will be present as a pair in the combined sample. Because these peptides are chemically identical, they will co-elute and will be visualized on the mass spectrum (m/z) at a predetermined mass distance of e.g. 8 Da. Relative expression of peptides is determined by ratios of the spectrum intensity of peptides. Thus, the process provides relative quantification of the change in abundance of protein expression at the global level in a perturbed, experimental state with respect to the control. Originally, the lighter linker contained no deuterium (only protium, i.e. ^1^H_1_) while the heavier linker had 8 deuterium atoms of hydrogen, thus providing a mass shift of 8 Da, as mentioned before. On the realization that deuterium can cause a large chromatographic shift on the virtue of reacting with the stationary phase of the chromatographic column, in later experiments, stable isotopes of carbon (^13^C_6_) were incorporated [19], as an improved and improvised version. One major drawback posed by this method was that it was biased against proteins lacking cysteine residues; only cysteine-containing proteins are identified and quantified. Studies have shown that proteins lacking cysteine residues could make up at least 10% of the total protein. Eventually, the method has been replaced with other, newer, and improved methods of labeling for quantitative proteomics, such as SILAC, iTRAQ, and TMT, as described below.

#### Tandem mass tags and iTRAQ—chemical labeling

*In vitro* chemical labeling of the proteome has many advantages and is the only option in cases where metabolic labeling cannot be used. The use of ICATs, however, comes with a few drawbacks, such as missing out on proteins that lack cysteine, and the propensity of deuterium to cause a chromatographic shift as described in the previous section. A heavier peptide labeled with deuterium elutes earlier than the lighter peptide; therefore, one needs to allow for the lagging peptide also to elute if accurate quantification is to be done. In doing so, a compromise on the accurate identification of the peptide (therefore, of the protein) by the MS/MS fragmentation is made. There is also a possibility that the lagging peptide of a peptide pair co-elutes with a peptide of another pair, thus increasing the possibilities of cross-quantification or suppression of ionization. To circumvent these limitations and have a sound chemical labeling strategy, another method for *in vitro* chemical labeling of the proteome was introduced by [20,20]. This method introduced a novel class of reagents termed tandem mass tags (TMTs) to isotopically labeled peptides of the sample. Akin to other labeling methods, TMT-tagged peptide pairs are also chemically identical and co-migrate in the chromatography column. The term tandem refers to the fact that, unlike other methods that quantify peptides at the MS1 level, labeling with TMTs allows both relative quantification as well as identification at the MS/MS (MS2) level. The quantitative measurements at the MS2 level have a better signal-to-noise ratio, thereby, using TMTs leads to more accurate relative quantification. In contrast to other methods, after the addition of TMT tags, peptide pairs have the same net molecular mass and are therefore known as isobaric tags. The TMT-tagged peptide pair is observed as only one peak in the MS1 scan. A TMT is made of three elements: a mass reporter, a mass normalizer (spacer), and an amine-reactive group. Different TMTs differ in the molecular mass of the reporter element, which is released on fragmentation in the MS/MS and used for relative quantification; the more abundant a protein in the sample, the higher the intensity of its reporter ions generated in the MS/MS spectra. Several TMT reagents have been developed and are commercially available: TMT-zero, TMT duplex, TMT 6-plex, TMT 10-plex, and TMT 16-plex. All of these have the same structure, but their reporter groups are made up of different numbers and combinations of stable isotopes of 15N (nitrogen) and 13C (carbon). To balance the total mass, the balancer group is varied accordingly, using isotopes. A TMT-6plex, e.g., would generate reporter ions in MS/MS which will appear at m/z of 126, 127, 128, 129, 130, and 131. Both iTRAQ and TMT usage involve the same principles for relative quantification. Use of isobaric tags provides several advantages over other labeling methods, such as greater multiplexing, better accuracy, and robustness, making them a preferably used method in the field of global proteomics.

#### Stable isotope labeling with amino acids in cell culture—metabolic labeling

In such metabolic labeling, stable isotopes are incorporated before the start of the experiment. Stable Isotope Labeling with Amino acids in Cell culture (SILAC), introduced by Shao-En Ong et al. in Mathias Mann’s group in 2002, was the first method to metabolically incorporate stable isotopes in the proteins for the purpose of proteome quantitation [21]. To metabolically incorporate stable isotopes with the aim of differentiating one proteome from another, one needs to change the way the subject grows and synthesizes its proteins. The procedure under SILAC involves labeling the building blocks of the proteins, i.e., amino acids, by replacing the naturally abundant isotopes with their heavier, stable isotopes in the growth medium. Preparation of the growth media is the only extra step required to make proteomics analysis quantitative in nature, via SILAC. To conduct the experiments, cells from two states (or more) to be compared are grown in different SILAC media, viz., light, medium, and heavy. These different media differ in their compositions in the isotopes of C, H and N atoms that form essential amino acids such as arginine and lysine amino acids. At the least, there can be three different media depending upon different combinations of stable isotopes in them (however, with different combinations and permutations, eight different conditions can be analyzed, provided that there is at least a 4 Da mass difference between the lighter and heavier versions of the peptide). ‘Light’ media contains Arginine and Lysine with normal 12C and 14N, and 1H. ‘Medium’ media contains six C atoms of Arginine made up of 13C and Lysine with four atoms of 2H. ‘Heavy’ media contains Arginine with six atoms of 13C and four atoms of 15N, and Lysine with six atoms of 13C and two atoms of 15N. Cells are adapted to the media of choice and subsequently allowed to grow for 5–7 doublings for optimum turnover of total protein in each media that facilitates maximum labeling of the proteome with their respective labels. After 5–7 doublings, the cell lysate is prepared and tested by MS analysis to ensure that label incorporation is more than 95%. Since the labeled amino acids are chemically identical to the naturally utilized ones, cells grown in media with isotopes are unaffected and normal, distinguishable only by MS [22]. Postlabeling, experimental perturbation is applied for the requisite time duration, cells are combined, harvested for the proteome, and digested with a site-specific protease like trypsin. Each peptide, thereon, will be present in a pair which will be chemically identical but different in abundance and will co-elute from the LC-column into the MS. The pairs on the mass spectrum will be as many Daltons apart as the extra mass added by the stable isotope. The ratio of the height of peaks of the pair gives the read-out of the relative abundance of the protein in the samples under comparison. SILAC can be used for any actively growing cell culture, including primary culture [23].

### Role of proteomics in cancer research

Studying proteome level changes with various methodologies and technologies as described above in cancer patients’ derived biospecimens aims to identify biomarkers that, with utmost sensitivity and specificity, reveal significant insights into the disease state. This has proven to be pivotal for understanding mechanisms, including rewired cellular signaling pathways that drive a particular cancerous pathology. While many cancer types have been classified and subclassified based upon their genomic and transcriptomic profiles to design targeted therapies, however, the functional relationship between RNA and protein products is modest. Cellular and molecular heterogeneity exists beyond DNA or RNA profiles, and therefore, it becomes pertinent to evaluate proteomic profiles, which reflect the functional cellular state, especially while identifying relevant biomarkers and designing targeted molecular therapies [24]. In addition to the fact that each tumor is a heterogeneous system made up of differently regulated cells, cancer cells originating from within the same tissue can manifest vastly different etiologies in different individuals. Characterizing features of heterogeneity via proteomics opens up new avenues to overcome resistance encountered against various anti-neoplastic agents and progress toward a personalized therapy.

### Understanding biological processes relevant to cancer progression (metastasis)

Metastasis, driven by the interaction of multiple factors in a coordinated manner, is a key hallmark of cancer, disease progression, and the leading cause of death in cancer patients [25]. Understanding cellular and molecular mechanisms of metastasis is therefore of utmost clinical importance.

The first major application of MS-based quantitative proteomics in cancer biology was to quantitatively study the proteome of pancreatic cancer via ICAT [26]. Differential expression of proteins such as annexin A2, cathepsin B, and matrix metalloproteinase, which help tumor invasion and metastasis by degrading the extracellular matrix, was observed. Similarly, proteome comparison of differently metastatic cell lines from cancers like prostate and colorectal cancer revealed proteins such as HSP27 and cofilin-1 (cf.L1) as potential mediators of metastasis [23,27]. cf.L1 seems to promote metastasis by remodeling the F-actin cytoskeleton and by activating P38 MAPK signaling pathway [28].

In order to metastasize, a tumor cell must degrade the local ECM components, which maintain a tissue in intact shape and structure. Proteases play a crucial role in clearing these physical obstructions for migration and toggle switches of the signaling circuitry. Of these proteases, matrix metalloproteinases (MMPs), especially MMP2 and MMP9, are essential for metastasis and angiogenesis [29]. Incomplete knowledge of their substrates has been a reason for failure to overcome side effects during clinical trials, and consequently, the failure of broad-spectrum metalloproteinase inhibitors [30]. Therefore, understanding the roles of MMPs and their specific substrates is an important pharmacological target [31]. With the aim of deciphering how these proteases modulate signaling networks and, consequently, the cellular response in a pathological versus a normal physiological state, MS-based quantitative strategies have been utilized to systematically discover the substrates of these MMPs and measure changes in their abundances in cancer.

Using the method of iTRAQ, Dean and Overall (2007) identified novel substrates of MMP2. These novel substrates, which were also subsequently biochemically corroborated, included osteopontin, HSP90α, Galectin-1, and CX3CL1 chemokine fractalkine (Dean RA and Overall CM. 2007). This was the first study to use iTRAQ for studying cell-conditioned medium for protease substrates. Novel substrates of MMP9—a key metalloproteinase—were identified by [32,32] using label-free quantitative proteomics to understand its contribution to the process of metastasis. In addition to metastasis, MMP9 is known to contribute to cancer progression and activation of angiogenic switches. Silencing of MMP-9 in tumor cells leads to reversion to a less metastatic phenotype in breast cancer cells [33–35]. Similarly, six new substrates of MMP9 were identified: APP, PCDGF, PN-1, Collagen VI, LIF, and Neuropilin-1, and further validated using immunoblotting [31]. In addition to differentially expressed proteins, proteolytically processed states of a proteome (proteins) can also be considered as biomarkers to identify diseased states vs. normal. Proteomic studies have also revealed that cells with high invasive potential tend to express more Epithelial-to-Mesenchymal Transition (EMT) related proteins. It was observed in a deep proteomic study of triple-negative breast cancer (TNBC) cell lines by Lawrence et al. [36] that most invasive TNBC cell lines had lowered expression of proteins involved in cell proliferation and heightened expression of proteins involved in migration and epithelial-to-mesenchymal transition [36]. This also implied that comprehensive proteome analysis, when coupled with intelligent bioinformatic analysis, can reveal clinically useful insights about the molecular behavior of recalcitrant and malignant cell lines such as TNBC, as well as many other cancers. Such comprehensive understanding of functional proteome changes opened many promising avenues of development of new drugs that target the most important of cancer hallmarks, such as metastasis.

### Finding novel biomarkers in a quest for stratifying heterogeneous cancers

Identifying cells diseased from a milieu of normal cells requires identifying information or an inventory of features/attributes/traits, or its various manifestations present in the body characteristic of their existence, to help distinguish them from each other. Such quests for molecular biomarkers date back to as early as 1847 when the first cancer biomarker was identified by the English physician Henry Bence-Jones. Jones discovered a large quantity of a monoclonal globulin protein - ‘Bence Jones Protein’ in the urine of patients with multiple myeloma. It was later found to be an antibody light-chain produced by the tumor, and this protein is present in the serum as well [37–39]. Based on their usage, biomarkers can be categorized as diagnostic markers, prognostic markers, predictive markers, and monitoring markers. The source of such biomarkers can be tissue biopsies, circulating tumor cells, or body fluids like blood, serum, plasma, sweat, urine, and, recently, breath condensates. These markers have an important role in helping clinicians to stratify patients into different risk groups and counsel them accordingly [20]. In principle, a reliable molecular biomarker must be both specific and sensitive. Analogically, reliable biomarkers are akin to fingerprints—unique, distinctive attributes that typify a biological state. In cancer, these indicators or biomarkers can function as read-outs of progressive precancerous and cancerous states to help identify the type and characteristics, predict progression and survival, essential for healthcare providers as guides to choose or devise appropriate treatment regimens. The main objective of finding biomarkers is to develop a noninvasive method of calculating risk and the earliest possible diagnosis. Currently, these functions are majorly fulfilled by the TNM classification system developed in 1957 by the AJCC [40]. The TNM classification system is based on the anatomical features of cancer. TNM classification has long been used successfully in local therapies such as surgical resection and/or regional radiotherapy. However, we now know that anatomically similar-looking cancers may be driven by very different key molecular trademarks. Even cancers with equivalent TNM staging may result in unexpected and varied therapeutic outcomes. There is an urgent need for molecular biomarkers which can accurately reflect the cancer characteristics to help stratify heterogeneous cancers into further subtypes for a tailored and effective treatment and management. Successfully validated molecular biomarkers are being explored for use in conjunction with the formal TNM staging. This is exemplified by research on various cancers. Proteomics and phosphoproteomic analysis have been utilized to subcategorize esophageal cancer to enhance therapeutic targeting [41]. Proteome profiling of such subtypes can help stratify these subtypes and reveal subtype-specific vulnerabilities which can be exploited to develop more effective therapeutic regimens [42]. Such knowledge, for instance, can be crucial in deciding whether to choose chemotherapy or immunotherapy for a particular subtype. Mouse models that recapitulate the pathobiology of human cancers have also been used to discover cancer biomarkers. Proteins whose abundance changes as the tumor develops can be identified using xenograft models of human cancers, such as in orthotopically implanted lung cancers in mice [43,44]. These studies may lead to the discovery of potential biomarkers applicable to humans for noninvasive diagnosis.

Proteins secreted by tumor cells are also windows into diseased states of cells. The secreted tumor proteins can be found in patient sera and other body fluids. Analyzing the proteome of urine samples from 231 humans, which consisted of healthy individuals and individuals with benign pulmonary disease, lung cancer, cervical cancer, colorectal cancer, or bladder cancer, Zhang et al. [45] could identify a panel of five biomarkers: FTL (ferritin light chain), MAPK1P1L (mitogen-activated protein kinase 1 interacting protein like), FGB (fibrinogen beta chain), RAB33B (RAB33B, member RAS oncogene family), and RAB15 (RAB15, member RAS oncogene family). A combination of these could not only diagnose healthy individuals with lung cancer but also distinguish lung cancer patients from patients with other forms of cancer [45]. The secretome of cancer cell lines can be a proxy for studying signature proteins secreted by tumor cells. The MS study carried out by Planque et al*.* analyzed the proteome of conditioned media from four lung cancer lines of different histological backgrounds and identified five novel proteins: ADAM-17, pentraxin 3, osteoprotegerin, follistatin, and tumor necrosis factor receptor superfamily member, 1A as promising as diagnostic biomarkers for lung cancer [46]. Similar proteome studies in lung cancer have identified dihydrodiol dehydrogenase (DDH) and L-lactate dehydrogenase B as potential candidate markers, which are secreted in patients’ sera by tumor cells of the lung [47]. Bottom-up proteomics via SILAC conducted by Yocum et al*.* on leukemia cell lines (MV4-11 and RS4;11) observed that decreased expression of nm23 can provide information on the extent of inhibition of HSP90, and therefore on the efficacy of the ongoing treatment [48].

To fully understand the intricacy of cancer, more molecular events of the disease need to be unraveled. Advanced omics technologies have been quite useful in this pursuit, where both proteome and phospho-proteome analyses have made it easy to discover several new biomarkers and drivers of tumor progression. Recently, researchers identified previously uncharacterized genes, transmembrane protein 79 (TMEM79), and ACOXL1, as potential diagnostic markers for prostate cancer using patient-derived prostate-specific proteome [49]. This proteome analysis was done in conjunction with assay data from RNA-sequencing, which provided a prostate-specific transcriptome and antibody-based protein profiling and indicated significant correlations of the RNA and protein expressions for each gene. Differences in the protein expression profiles between normal and prostatic cancer tissue then serve to further endorse the potential of these genes as ideal biomarkers for early prostate cancer detection [49]. Ecker, Mark A. et al. used a label-free proteome workflow to examine ovarian cancer progression using 11 high-grade serous ovarian cancer (HGSC) patients’ cohort and identified nicotinamide N-methyltransferase NNMT as a key metabolic regulator of cancer-associated fibroblasts (CAF) phenotype differentiation and cancer progression, suggesting a potential therapeutic target [50]. Often, biomarkers that are ideal for diagnosis or prognosis can also be good targets for pharmacological intervention.

### Development of novel cancer therapies

Proteomics-based studies have successfully contributed to the discovery of molecular targets overexpressed in cancers. Molecular therapeutic interventions, especially in the case of TNBCs, have not been successful, for there has been no recurring genetic aberration identified for it. To this end, Cabezon et al*.* studied the proteome of 78 TNBC samples and identified *Mage-A4* as a potential therapeutic target. This finding is significant as the protein is not only present in tumor lesions but is also detectable in interstitial fluids and sera of the patients [51]. Proteomics experiments have also helped develop knowledge to discriminate between secondary consequences of primary cancer. For instance, head-and-neck small cell carcinoma (HNSCC) frequently leads to metastases in the lung (metHNSCC). The patients of HNSCC also tend to develop (primary) metachronous squamous cell carcinoma of the lung (SQCLC). A successful therapeutic intervention warrants successful discrimination of the two; therefore, it is crucial to have signature molecular features available from each. To pursue this, a comparative proteome analysis of 42 SQCLC and 30 HNSCC tumors conducted by Bohnenberger et al*.* revealed their distinct expression profiles carrying signatures of their genetic origin [52]. Comprehensive validation of such molecular signatures in patients with tumors of unknown origin helps in their prognostic segregation, thus in tailoring therapeutic regimens accordingly in a near-personalized manner.

Lung cancer leads to the maximum number of cancer-related deaths worldwide [53]. Current diagnosis for lung cancer mainly relies upon invasive methods combined with imaging, and mostly, the patients get diagnosed when the tumor has reached advanced stages. Thus, molecular methods that help early diagnosis, identify successful therapeutic targets, and, consequently, improve survival rates are warranted. It was realized only after 2004 that different subtypes respond better to subtype-specific therapies; therefore, stratifying tumors based on their genotype or expression profiles is crucial [54]. Tumor tissue and its paired noncancerous adjacent tissues (NATs) follow different projections of development and therefore are governed via different signaling pathways activated in them. Proteins such as AGER, CACNA2D2, LAMP3, SCGB1A1, SFTPA2, SFTPB, and SFTPC, which are associated with normal lung physiology, tend to lose their expression in the tumor tissue [55,56]. Identification of relevant substrates of MMPs in particular cancer and then designing inhibitors specific to them would prove to be effective anti-cancer strategies, with the least side effects; the method of MS-based degradomic studies in cataloguing them along with their abundance is a comprehensive method.

As the disease progresses, the tumor often tends to become increasingly heterogeneous, both between individuals and within a tumor. An outcome of such heterogeneity is the establishment of diversity of cancer cells, which differ in their sensitivity to treatments [57]. Studying heterogeneity helps understand why treatments that are targeted specifically against a classical subtype (and therefore are expected to have favorable outcomes) often fail, and the cancer tends to relapse. Addressing such extensive heterogeneity among cancers requires the classification of cancers into distinct, nonoverlapping categories based on their molecular signatures. In-depth proteomic analysis of four primary tumors and a panel of 20 different cell lines derived from breast cancer patients to understand heterogeneity within the classically defined subtype—TNBC—helped redefine subtypes based on differential protein expressions. The changes in protein expressions were also correlated with mutations in exonic regions [36]. A similar study analyzing proteomes of 102 paraffin-embedded breast tumor samples, which were classified either as estrogen receptor (ER)+/progesterone receptor (PR)+or triple-negative, revealed that some of the ER+/PR+tumor samples shared expression profiles with those of triple-negative cases [58]. Knowledge of such expression profiles is a valuable addition to existing standard tests in terms of prognosis and substratification of conventional subtypes. Particularly new knowledge base of targets for drug screening. Proteomics of TNBC tissues compared with normal breast tissues by Muñiz Lino MA et al. revealed at least 12 proteins (COX5, DB1, DJ1, eIF5A-1, FTL, HSP27, MTPN, PSME1, RhoGDI-2, SH3BGRL, SOD1, and UBE2N) to be significantly over-expressed; of these, COX5, DB1, and MTPN were never reported before and, thus, presented as new candidate biomarkers of TNBC [59].

Intratumor heterogeneity is fueled either by genetic or microenvironmental drivers. Cancer cells, in response to hypoxia and to meet the demands of oxygen, make either imperfect attempts to form new blood capillaries or attempt to change the tone of existing capillaries i.e., dilation of blood capillaries and vessels. This adaptation program leads to the activation of gene sets involved in glycolysis, metabolism, angiogenesis, cell proliferation, differentiation, and apoptosis [60]. The results of these re-adaptation strategies are manifested as increased metastatic potential, aggressiveness, invasiveness, and increased resistance toward chemotherapy and radiotherapy. Proteome comparisons to study hypoxia-induced changes revealed overexpression of exosomal proteins, which are associated with metastasis and angiogenesis [61]. Utilizing the method of multiple reaction monitoring (MRM) to validate proteomics results in epithelial carcinoma revealed upregulation of Ku70/Ku80 dimer—key proteins in the nonhomologous end-joining pathway in response to hypoxia [62].

Discovery of biomarkers has resulted in more effective therapy and targeted drugs such as imatinib (Gleevec) [63], gefitinib (Iressa) [64], and cetuximab (Erbitux) [65]. A biomarker may represent multiple tumor types, as all cancers fundamentally involve deregulated expression and signaling pathways [66]. In this regard, the use of a panel of the combination of informative markers has been suggested. Through extensive collaborative clinical and molecular research, numerous candidate molecular markers have been identified. However, due to factors ranging from lack of rigorous clinical validations to regulatory approvals, there are only a few that have been brought into clinical practice. For instance, many candidates, when tested in large-sample studies, show poor diagnostic value, or low specificity and sensitivity [67]. Out of 1261 candidate biomarker proteins, only 41 are used for clinical purposes, and fewer are FDA-approved. Some selected ones that are approved by the FDA and are in clinical use include Pro2PSA, ROMA, Ova1, p63 protein, CA19-9, α-Fetoprotein, β-HCG, LDH, Her2/Neu, and CA125 [68]. The next-generation proteomics technologies have opened up many bottlenecks hitherto posed by low-abundance candidate biomarkers, which escaped the detection limits of earlier available methods.

### Overcoming chemoresistance

Despite advances in radiotherapy, chemotherapy, and immunotherapy treatments, recurrence persists as a significant cause of mortality. Identification and characterization of therapy-resistant cells within the tumor heterogeneous microenvironment have drawn attention in the past decade [69–72]. Proteomics analysis has been instrumental in identifying new molecular targets responsible for acquired therapy resistance in cancer cells.

For instance, in the case of pancreatic ductal adenocarcinomas (PDAC), the generation of gemcitabine-resistant cells confers aggressiveness to the disease and, thus, decreases survival rates. In that respect, Le Large, Tessa Ya Sung et al. [73] investigated the proteome and phosphoproteome of those gemcitabine-resistant cells and identified a microtubule-associated protein, MAP2, as an up-regulated protein associated with acquired resistance. Surprisingly, Map2-expressing gemcitabine-resistant cells were found to be sensitive to Nab-paclitaxel, an alternative chemotherapy drug [73]. This study highlighted the clinical benefits of combination therapy, particularly for gemcitabine-resistant PDAC, and emphasized the potential of leveraging MAP2 as a therapeutic target [73]. Proteomic and metabolomic analysis of carboplatin-resistant ovarian cancer cells identified the overexpression of FABP4, a lipid chaperone protein, as a crucial regulator of lipid responses in ovarian cancer cells that interact with adipocytes recruited by cancer cells during metastasis [74]. FABP4 knockdown down-regulated the signature genes involved in ovarian cancer and even decreased the clonogenic survival rate. Moreover, inhibition of FABP4 by its small molecule inhibitor, BMS309403, reduced the tumor burden and highly increased the sensitivity of ovarian cancer cells to carboplatin, both in vitro as well as in vivo [74]. Such results from proteomic analyses have led to the discovery of new targets responsible for drug resistance in cancers and even suggested a strategy to maximize the effectiveness of usage of existing chemotherapy by the inclusion of additional drugs that target proteins involved in drug resistance.

### Critical contribution in the development of immunotherapy

Immunotherapy based on immune checkpoint inhibitors (ICIs), such as anti-CTLA4, anti-PD1, and anti-PDL1, has been a game-changer in cancer treatments. The process involves directly boosting the production of tumor-reactive T-cells, which generate a natural defense against cancer cells inside the patient’s body [75,76] or an alternative strategy of adoptive cell therapy (ACT), such as genetically engineered T-cells expressing tumor-recognizing receptors (CAR-T) or tumor-infiltrating lymphocytes (TIL)- derived T cells [77,78]. Although immunotherapy has shown promising results in many cases [79,80], there have been reports of failure either by primary resistance due to lack of response or through acquired resistance after initial response [81]. Intrinsic as well as extrinsic factors contribute to both primary and acquired resistance to immunotherapy [82]. While tumor intrinsic factors are due to inability of immune recognition as observed with downregulation of PD-L1, an immune receptor induces T-cell anergy [83,84], down-regulation of antigen-presentation systems, and major histocompatibility complex (MHC) presentation, dysregulation of oncogenic pathways like p53, RAS/RAF/MAPK signaling and others; tumor extrinsic factors are due to resistance within tumor microenvironment (TME) and toward immunotherapy [84]. Through these and other examples, proteomics-based analyses have contributed in compelling ways to give a deeper understanding of immunotherapy response. The utility of MS-based proteomic studies extends to different biological samples such as tumor biopsies, liquid biopsies—plasma, blood, serum, or other fluids, and TME derived cells isolated using fluorescence-activated cell sorting (FACS). Detailed proteome profiling can help in decoding new biomarkers that can predict the immunotherapy response and give insights into molecular and biological changes induced post-therapy [85]. To achieve this, deep proteomic profiling was first done by Harel, Michal, et al. [86] to study immunotherapy response in advanced-stage melanoma patients. Proteome analysis of 116 patients divided into two cohorts, one treated with TIL-based and another with anti-PD1 therapy, revealed higher lipid metabolism and oxidative phosphorylation in responders as compared with the ones not responding to the treatment. This high mitochondrial metabolism is associated with higher antigen presentation and IFN signaling, thus increasing T-cell-based killing of cancer cells both *in vitro* and *in vivo* [85,86]. In continuation, the team performed MS-based proteomics analysis of 185 formalin-fixed, paraffin-embedded samples originating from advanced metastatic melanoma patients given TILs, anti-CTLA4, or anti-PDL1 therapy. Proteome profiling along with bioinformatics analysis revealed the association of tumor molecular profiles with metastatic location, BRAF mutation status, immunotherapy response, metabolic processes, and overall survival. The study revealed the clinical importance of molecular profiling in metastatic melanoma, including BRAF mutation status and prior treatments with MAPK inhibitors, in determining immunotherapy response and suggesting combination therapies for increased survival [87]. Another important aspect of tumor immunity studies is to identify antigens present on the surface of tumor cells. This information is utilized to develop patient-tailored immunotherapies like CAR T-cell therapy and personalized vaccines. MS-based proteomics strategies, including ion mobility separation-based time-of-flight (TOFIMS) MS, are utilized for immunopeptidomics where tumor antigen identification is done by analyzing peptides bound to human leukocyte antigen (HLA) expressed on tumor cells [85,88,89].

### Harnessing the scope of liquid biopsy for early detection and biomarker discovery

Proteome analysis poses a significant challenge compared with genome analysis due to its inherent dynamicity. Unlike the relatively stable genome, the proteome exhibits variations due to the changing molecular landscape of a cell. Despite this limitation, proteomics has emerged as a preferred technology in the pursuit of discovering novel biomarkers for improved and early diagnostics [90]. Liquid biopsies have great potential in the areas of early detection of cancers, diagnostics, prognostics, and the real-time monitoring of patient responses to therapies and relapses in the area of oncology and precision medicine. Proteome analysis is itself a rapidly advancing research area in cancer liquid biopsies [66,91–93]

Liquid biopsy, with a noninvasive or minimally invasive mode of sample collection, has shown tremendous value in the early diagnosis and monitoring of cancers, especially with hard-to-access surgical tumor biopsies. In uveal melanoma, an eye tumor where 50% of all patients develop metastatic disease, of which 85% die because of visceral metastasis; tumor biopsies are mandatory for identifying individuals who are prone to develop metastatic stages. However, tumor biopsies also carry the risk of retinal detachment and tumor dissemination, making them less preferable. In this regard, a proteome study of liquid biopsies from uveal melanoma (UM) vitreous has proven to be very beneficial. Gabriel Velez et al. (2021) revealed that increased expression of HGF, HGFR, and SCFR, along with decreased expression of KLK7, is associated with metastatic uveal cancer [91].

Proteome analysis of serum samples from patients has been consistently utilized for elucidating disease biology and therapeutic options [94–98]. Utilizing LC coupled with MS/MS analysis in comparison with healthy controls, as performed in a gastric cancer study, could not only identify disease-specific proteome but also quantify several proteins potentially implicated in gastric cancer. Abundant serum proteins like albumin, haptoglobin, and transferrin are initially removed using multiple affinity removal systems and Cu column chromatography in this protocol. Subsequently, LC ESI-MS/MS was employed to discover proteins associated with gastric cancer. The study employed a label-free approach of MS to quantify differentially expressed proteins in gastric cancer, and through multiple reaction monitoring analysis, four proteins—Clusterin isoform 1 (CLU-1), thrombospondin 1 (TSP1), tyrosine-protein kinase SRMS, and Vitronectin (VTN) —were identified as serum biomarkers capable of differentiating gastric cancer patients from the normal population with significant specificity and selectivity [99].

Kim, Y. et al. (2016) focused on utilizing targeted proteomics through selected reaction monitoring-mass spectrometry (SRM-MS) and liquid biopsy techniques to develop liquid-biopsy signatures for accurate patient classification. They aimed to verify candidate biomarkers identified through high-resolution proteomic technologies and evaluate the feasibility of targeted proteomic assays using expressed prostatic secretions (EPS) urine (a tissue-proximal fluid) as a clinically applicable sample. The study demonstrated the potential of SRM-MS in quantifying and verifying biomarker candidates, narrowing down the candidates to 34 peptides from over 200 candidates with high biomarker potential to distinguish prostate cancer patient risk groups [93].

Circulating tumor cells (CTCs) were classified as stable or unstable based on their EMT phenotype, which provided insights into clinical prognosis [100–102]. The plasticity of CTC phenotypes contributes to cancer metastasis, suggesting their value as early metastatic event indicators, and EMT CTCs exhibited enhanced metastatic abilities and resistance to chemotherapy [103]. Huang, Mao, et al. [104] highlighted the diagnostic and therapeutic potential of proteomics in CTCs. In this study, the proteomic analysis revealed significant changes during CTC metastasis and identified EMT-related protein clusters associated with the regulation of the pathways involved in metastasis, EMT, and immune cell infiltration. ERBB2, CAVIN1, and COL6A were identified as potential biomarkers and therapeutic targets for early metastasis [104].

### Single-cell and spatial proteomics as promising allies of cancer therapy

MS-based proteome profiling has advanced to provide increasingly comprehensive and quantitative data, with optimal results achieved from high sample concentration. This allows for an in-depth measurement; however, the larger the sampling size, the higher the number of cells. Tumors and cancers exhibit cellular heterogeneity, and often, poorly represented smaller subsets of cell types are ignored in the analysis. With increasing evidence of small subsets or niches, such as cancer stem cells, driving the progression and therapeutic response of cancer cells, proteome community sought to find ways to capture cell-specific information. Development of single-cell proteomics has opened up new avenues for a granular understanding of biological systems [105]. Single-cell proteomics offers the potential to reveal how combined actions of multiple gene products drive specific cellular phenotypes, providing a deeper mechanistic understanding of cellular function. This advancement has augmented our understanding of protein expression in a diseased/ tumor cell manifold. It addresses the singular bottleneck of intratumor heterogeneity by providing valuable insight into the composition of each cell type and its prognostic value. MS-based single-cell studies like the Single-Cell ProtEomics technique (SCoPE-MS) are unbiased proteomics profiling methods promising to link protein expression with phenotypes of interest, to study tumor heterogeneity [106]. Along with the single-cell resolution of a heterogeneous population of cells in a tumor with minimal or no protein loss, the quantification of single-cell peptides can also be achieved. Single cells are microscopically picked and mechanically lysed to avoid any loss, whereas the quantification of peptides is done using tandem mass tags. A recent study demonstrated a microscopy-based SCoPE-MS proteome profiling that performs real-time monitoring and obtains intracellular dynamics analysis to discover key proteins that contribute to abnormal DNA damage response [107]. Characterization and stratification of cell types through single-cell resolution analysis are some of the exciting areas of current research in this field.

Single-cell proteomics gives the opportunity to examine functionalities of proteins and their interactions at a single-cell level; however, it does not correctly perceive the interaction between different cells with their individual cellular and molecular attributes. This lacuna is resolved by including spatial analysis, an arrangement of cell types in time and space, in the pursuit of understanding heterogeneity. It was observed that variable functionalities of the same proteins with respect to their spatial attributes could be captured, providing invaluable information on how proteins behave in different locations of cells and tissues. For instance, β-catenin, a multifunctional protein, is oncogenic when present in the nucleus and cytoplasm, whereas it is tumor-suppressive when present in the plasma membrane [108,109]. As a potential therapeutic target, information on such proteins through spatial proteomics, along with single-cell proteomics, will provide researchers with information that can allow more precise targeting of the drug.

At the cellular front, novel stratification of cell types such as cancer-associated fibroblast (CAFs) has emerged, where nine distinct CAF types were identified in breast cancer using single-cell RNA sequencing data and validated the data through multiplex mass cytometry and spatial proteome distribution of defined marker genes of each subtype in patient samples [110]. Li, Meiyi et al. revealed that nonalcoholic steatohepatitis hepatocellular carcinoma (NASH-HCC) resists immune checkpoint blockade due to its unique tumor immune microenvironment, characterized by spatially heterogeneous immune cell infiltration. This study revealed interactions between myeloid-derived suppressor cells, exhausted T cells, and tumor-associated macrophages as key to its immunosuppression, offering potential therapeutic targets [111]. Sorin et al. employed imaging mass cytometry to analyze samples from 416 LUAD patients, identifying over 1.6 million cells and revealing distinct immune lineages and activation states correlated with clinical outcomes [112]. The same group performed a spatial landscape of immunotherapy response, revealing that CXCL13 plays a crucial role in enhancing antitumor immunity, particularly in the context of immune checkpoint inhibitors (ICIs) in lung cancer [113]. Single-cell and spatial proteomics have advanced significantly in recent years; some of these are listed in Table 1, with notable improvements in resolution, sensitivity, and data analysis capabilities [114–122]. These advancements have enabled deeper insights into cellular heterogeneity, tissue architecture, and protein dynamics, facilitating breakthroughs in fields such as oncology, immunology, and neuroscience.

**Table 1:** Recent advancements in single-cell and spatial proteomics.

Techniques	Description	Research	References
Zwitterionic detergent and catalyst-based SCP in a micro-HOLe disc (microHOLD)	A novel mass-compatible approach using a zwitterionic detergent and catalyst facilitates efficient single-cell lysis and TMT labeling within a micro-HOLe disc. This method minimizes peptide loss and enhances the accuracy of proteomic analysis.	Analysed proteome reprogramming in hormone-sensitive prostate cells as they transition to castration-resistant prostate cancer cells, demonstrating the technique’s practical utility in advancing cancer research.	[114]
Single-molecule unmasking via DNA- points accumulation for imaging in nanoscale topography (SUM- PAINT)	A high-throughput imaging method capable of achieving multiplexing at better than 15 nm resolution and visualizing up to 30 different protein types in a single sample—a comprehensive workflow for spatial proteomics	The study generated 30-plex single-molecule datasets in neurons and adapted omics-based analysis, uncovering synaptic heterogeneity and identifying a unique synapse type.	[115]
Cell painting with deep learning by cell morphological profiling (cmVIP)	Morphological profiling technique that utilizes six different stains to capture various features of cells and organelles, and incorporates deep learning algorithms to extract features from the images	The research predicted the functional impact of lung adenocarcinoma-associated somatic variants.	[116]
Deep visual proteomics (DVP)	An approach that integrates artificial intelligence with high-resolution single-cell or single-nucleus imaging, laser microdissection, and ultra-sensitive mass spectrometry to elucidate single-cell identity and heterogeneity.	Proteomic changes in melanocytes progressing to melanoma were identified and revealed alterations in spatially distinct pathways associated with cancer progression.	[117]
Chimeric antigen receptor (CAR)	A single-pass transmembrane receptor designed to target hematologic malignancies, achieving a high remission rate.	Primarily applied for hematological malignanciesCD19-CAR T cell activation triggers both CD19-CAR-specific pathways and canonical TCR signaling.	[118,119]
Quantification of RNA and intracellular epitopes by sequencing (QuRIE-seq)	High-throughput droplet-based platform to quantify single-cell mRNA and intra- and extracellular (phospho)proteins by sequencing within thousands of single cells.	Cell-state alterations at both the protein signaling and transcriptomic levels were detected after activation of the B-cell receptor pathway in Burkitt’s lymphoma cells	[118,120]
Single cell ProtEomics by Mass Spectrometry (SCoPE2)	SCoPE2 enhances quantitative accuracy and throughput while reducing costs and hands-on time through an automated, miniaturized sample preparation workflow.	Analyzed macrophage heterogeneity by quantifying over 3042 proteins in single cells, revealing continuous proteome state gradients during macrophage differentiation without polarizing cytokines.	[121]
Single-cell IsoCode chip	A highly multiplexed chip featuring an antibody barcode array enables the concurrent detection of proteins secreted by individual cells.	They decoded functional cellular heterogeneity among patients and provided predictions for clinical outcomes as well as potential toxicities associated with CAR therapies.	[122]

### Challenges in drawing conclusions from proteomics data in cancers

Proteomics has emerged as a very efficient way of investigating cancer as a disease. It has provided valuable insights into the underlying mechanisms of cancer development, progression, and treatment. However, due to the multi-layered regulatory landscape in cancers, drawing meaningful conclusions from proteomic data presents some challenges that will need to be addressed in the near future to improve accuracy and reproducibility.

Heterogeneity in tumor/cancer samples has been a formidable challenge, and proteome analysis has been at the forefront in attempting to stratify such cancers. Therefore, proteomics studies often involve the analysis of multiple samples from different patients to increase the statistical power and minimize the effect of individual variations. However, this approach can also bring in new sources of variability, such as differences in sample preparation, handling, and storage, as highlighted in studies by Mertins et al. (2016) and Liu et al. (2021) [41,123]. With this in mind, many researchers have used microdissection techniques to purify the specific cell populations from the tumor tissue. Regardless, these are complicated techniques for clinical laboratories and may not always be possible; therefore, relevant information may be lost within the process.

Irregularities introduced within the workflow at the point of sample preparation remain a major hurdle in single-cell proteomics concerning the reproducibility of data. Currently, nano-ProteOmic sample preparation (nPOP) [124] is utilized over minimal-ProteOmic sample preparation (mPOP), enabling the simultaneous preparation of 2000 single-cell samples for MS. This involves lysis, digestion, and labeling individual cells as droplets. However, the interaction of samples with plastic surfaces before MS detection leads to protein adsorption, which is detrimental for small samples like single cells ( < 1 ng). To minimize sample loss due to protein adsorption (PAL), specific detergents like n-dodecyl-ꞵ-maltopyranoside (DDM) are employed. DDM prevents protein binding to plastics without interfering with ionization and elutes out during washouts [125,126]. Some studies consider that the disparity in ionization efficiency poses a greater challenge than PAL. Sodium and phosphate ions in common buffers reduce droplet vapor pressure, lowering ionization efficiency by electron spray (ES). To address this, desalting or using volatile buffers such as ammonium acetate and ammonium bicarbonate proves effective [127,128].

Similar to other OMICS studies, proteomics presents a large volume of data to the scientific community. Proteomics studies often generate thousands or millions of data points, which can be difficult to manage and analyze using traditional statistical methods. Additionally, the dynamic range of protein abundance in biological samples has several orders of magnitude, making it difficult to quantify low-abundance proteins accurately. This data is often noisy and display technical and biological variations, which can further complicate data analysis. To address these challenges, researchers have developed a variety of statistical and computational methods for analyzing proteomics data, such as machine learning algorithms and network analysis approaches, to standardize the analysis approaches. However, these methods require careful consideration of the multiple testing problem and the selection of an appropriate significance threshold [129].

Nonuniformity/disparity in data analysis and data interpretation has been addressed in various research papers and editorials. The data generated from proteomics studies are complex in nature and need sophisticated analytical software and expertise in bioinformatics for interpretation. There is a lack of standardized data analysis workflows, and different methods can yield different results. Moreover, proteomics studies are still not cost- and time-effective and more often than not, involve a limited number of samples for analysis. This can result in low statistical power, with potential false-positive or false-negative findings. Thus, the biological relevance of the identified proteins needs to be validated through functional studies [130]

Notwithstanding challenges encountered as discussed above, MS-based proteomics remains crucial in understanding molecular phenotypes—metastasis, invasion, and heterogeneity in cancers. Since proteins are the main executors of cellular functions, and the transcriptome does not always correlate with the proteome of a given system, studying proteomics is indispensable to understanding biological systems, especially cancers, which are highly diverse and heterogeneous. Best practices and guidelines in the analysis of proteomics data in cancer research should consistently evolve to promote the optimum utility of the data. Standardization of workflows and methods of data analysis in the field of proteomics is, therefore, paramount to ensure uniformity and reproducibility of results between different studies and different platforms. Identification of protein biomarkers would be crucial for diagnosis, prognosis, and stratification of various cancer types. Systematic and structured proteomics studies must be carried out for different cancer types and subtypes, and data generated must be consolidated into well-organized databases. Combining clinical insights from the proteomics studies with insights from omics studies at different regulatory levels (multi-omics), such as transcriptomics and genomics, will lead to a finer resolution and stratification of cancer types, thus expanding the scope of personalized therapies. Proteomics continues to evolve at an extraordinary pace and has emerged as a strong technique to assist cancer researchers in their quest for biomarker research, along with remarkable progress in deciphering tumor biology.

## Open Access

This article has been published open access under our Subscribe to Open programme, made possible through the support of our subscribing institutions, learn more here: https://portlandpress.com/pages/open_access_options_and_prices#conditional

## Abbreviations

TNM: Tumor, Node and Metastasis
ACOXL: Acyl-CoA oxidase-like protein
ACT: Adoptive Cell Therapy
AJCC: American Joint Committee on Cancer
CAF: Cancer-Associated Fibroblasts
CAR: Chimeric Antigen Receptor
CI: Chemical Ionization
CTCs: Circulating Tumor Cells
DDH: Dihydrodiol Dehydrogenase
DDM: n-Dodecyl-β-Maltopyranoside
2-DE: Two-dimensional electrophoresis
2,5 DHB: 2,5-hydroxybenzoic acid
DIOS: Desorption/Ionization on Silicon
DVP: Deep Visual Proteomics
EI: Electron-impact Ionization
EPS: Expressed Prostatic Secretions
ESI: Electrospray Ionization
FACS: Fluorescence-activated cell sorting
HGSC: High-Grade Serous Ovarian Cancer
HLA: Human Leukocyte Antigen
HOLD: Micro-HOLe Disc
HPLC: High-Performance Liquid Chromatography
HSP90: Heat Shock Protein 90
ICAT: Isotope-Coded Affinity Tags
ICIs: Immune Checkpoint Inhibitors
IEF: Isoelectric focusing
LC-MS: Liquid Chromatography-Mass Spectrometer
MALDI: Matrix-Assisted Laser Desorption/Ionization
MHC: Major Histocompatibility Complex
MMPs: Matrix Metalloproteinases
MRM: Multiple Reaction Monitoring
MS: Mass Spectrometry
NATs: Noncancerous Adjacent Tissues
PDAC: Pancreatic Ductal Adenocarcinomas
POP: minimal-ProteOmic sample Preparation
POP: nano-ProteOmic sample preparation
QuRIE-seq: Quantification of RNA and Intracellular Epitopes by sequencing
SALDI: Surface-Assisted Laser Desorption/Ionization
SCoPE-MS: Single-Cell ProtEomics-Mass Spectrometry
SILAC: Stable Isotope Labeling with Amino Acids in cell culture
SRM-MS: Selected Reaction Monitoring-Mass Spectrometry
SUM-PAINT: Single-Molecule Unmasking via DNA-Points Accumulation for Imaging in Nanoscale Topography
TIL: Tumor Infiltrating Lymphocytes
TME: Tumor Microenvironment
TMEM79: Transmembrane Protein 79
TMT: Tandem Mass Tags
TNBC: Triple-Negative Breast Cancer
TNM: Tumor, Node and Metastasis
TRAQ: Isobaric Tag for Relative and Absolute Quantification
UM: Uveal Melanoma
